# No obesity paradox in out-of-hospital cardiac arrest: Data from the Swedish registry of cardiopulmonary resuscitation

**DOI:** 10.1016/j.resplu.2023.100446

**Published:** 2023-08-10

**Authors:** Alfred Hjalmarsson, Araz Rawshani, Truls Råmunddal, Aidin Rawshani, Clara Hjalmarsson, Anna Myredal, Gudrun Höskuldsdottir, Fredrik Hessulf, Geir Hirlekar, Oskar Angerås, Petur Petursson

**Affiliations:** a Institute of Medicine, Department of Molecular and Clinical Medicine, Sahlgrenska Academy, University of Gothenburg, Gothenburg, Sweden; b The Swedish Cardiopulmonary Resuscitation Registry, Västra Götaland County, Gothenburg, Sweden; c Department of Cardiology, Sahlgrenska University Hospital, Gothenburg, Sweden; d Department of Anaesthesiology and Intensive Care Medicine, Sahlgrenska University Hospital, Mölndal, Sweden

**Keywords:** Obesity, Diabetes, Out-of-hospital cardiac arrest, Survival

## Abstract

**Background:**

Although an “obesity paradox”, which states an increased chance of survival for patients with obesity after myocardial infarction has been proposed, it is less clear whether this phenomenon even exists in patients suffering out-of-hospital cardiac arrest (OHCA) and if diabetes, which is often associated with obesity, implies an additional risk.

**Objective:**

To investigate if and how obesity, with or without diabetes, affects the survival of patients with OHCA.

**Methods:**

This study included 55,483 patients with OHCA reported to the Swedish Registry of Cardiopulmonary Resuscitation between 2010 and 2020. Patients were classified in five groups: obesity only (Ob), type 1 diabetes only (T1D), type 2 diabetes only (T2D), obesity and any diabetes (ObD), or belonging to the group other (OTH). Patient characteristics and outcomes were studied using descriptive statistics, logistic, and Cox proportional regression.

**Results:**

Obesity only was found in 2.7% of the study cohort, while 3.2% had obesity and any type of diabetes. Ob patients were significantly younger than all other patients (p ≤ 0.001); the 30 day-survival was 9.6% in Ob, and 10.6%, 7.3%, 6.9%, and 12.7% in T1D, T2D, ObD, and OTH, respectively, with OR (95% CI) of 0.69 (0.57–0.82), 0.78 (0.56–1.05), 0.65 (0.59–0.71), and 0.55 (0.45–0.66) for Ob, T1D, T2D, and ObD, respectively (reference group OTH). No time-related trends in 30-days survival were found.

**Conclusion:**

Obesity was present in 6% of the population and was associated with younger age and a 30% reduction in survival; a combination of obesity and diabetes further reduced the survival rate.

## Introduction

The obesity pandemic is rapidly growing and in 2016 approximately 13% of the world’s population suffered from obesity, which is a twofold increase since 1980.^1^ Body mass index (BMI) is used to classify body weight, with BMI exceeding 30 kg/m^2^ defining obesity. Obesity increases the risk of multiple diseases, e.g. diabetes, hypertension, stroke, cardiovascular diseases, and several forms of cancer.

Out-of-hospital cardiac arrest (OHCA) is likewise a global health problem, with 360,000 individuals affected annually in the United States,^2^ and more than 500,000 individuals affected in Europe.^3^ Despite advances in resuscitation science, only 10% of victims of OHCA survive to hospital discharge in Sweden.^4^

Diabetes and obesity has been suggested as a deadly liaison that must be counteracted by all means available.^5^ As obesity is a risk factor for type 2 diabetes, these conditions often work in tandem. Furthermore, they are both complex and multifactorial, which make them harder to fully understand. Obesity and diabetes both significantly increases risk of cardiovascular diseases and stroke.^6^ However, there is sparse evidence of how patients with obesity versus diabetes differ in survival after an OHCA.

There is an overlap in risk factors and comorbidities in obesity and OHCA. Individuals with obesity have a higher risk of suffering a sudden cardiac arrest (SCA). However, the association between obesity and survival in SCA remains sparsely understood. Some studies show that obese people have the same or worse outcome, compared to non-obese individuals suffering an OHCA, while other studies suggest that obese individuals are more likely to survive, creating an “obesity paradox”.^7^, ^8^, ^9^ In recent years, however, the “obesity paradox” has been challenged by several studies.^10^, ^11^, ^12^, ^13^, ^14^

In the setting of OHCA, obesity poses a special challenge since it may affect both the underlying etiology of cardiac arrest as well as the efficacy of resuscitation and post-resuscitation efforts. This is especially important in an out-of-hospital setting as bystanders might not have appropriate knowledge to correctly perform CPR in these cases. We used the Swedish Cardiopulmonary Resuscitation Registry (SRCR) to study all cases of OHCA during 2010–2020 in Sweden. The primary focus of the study was to compare long- and short-term survival between five groups: obesity only, type 2 diabetes, type 1 diabetes, obesity and any diabetes, and all other cases. The secondary aim was to elucidate the clinical characteristics and differences in neurological outcomes between these groups.

## Methods

### Data sources

All emergency medical service (EMS) treated cases of OHCA in Sweden are reported to the SRCR. Data collection for OHCA was launched in 1990 and has been described in detail previously.^15^, ^16^, ^17^ Variables in the registry range from patient characteristics and spatiotemporal information to pre- and in-hospital management. The registry uses electronic data transfer and utilises the Utstein style of reporting.^18^ The data from SRCR was merged with data from the Longitudinal Integrated Database for Health Insurance and Labor Market registry (LISA), the Swedish Population Register, the National Prescribed Drug Register, to obtain comprehensive information about the study population. Information regarding the cause and date of death was retrieved from The Swedish Cause of Death. Data was obtained for both inpatient- and outpatient care; however, all in-hospital cardiac arrests were excluded.

We categorised patients into five groups: (1) obesity only (Ob), (2) type 2 diabetes (T2D), (3) type 1 diabetes (T1D), (4) obesity and any diabetes (ObD) and (5) all other (OTH). These diagnoses were based on the ICD (International Classification of Diseases) codes. Before the introduction of ICD-10 (1st of January 2011), ICD-9 codes were used. These codes were converted to ICD-10 codes after the change. ICD-10 codes were retrieved from the Swedish Inpatient and Outpatient Registry, which has complete level of ascertainment of inpatient care (since 1987) and outpatient care (since 2002). Obesity was reported as a diagnosis and no actual BMI-values were reported.

To assess the neurological outcome, we used the cerebral performance category score (CPC score). CPC score ranges from 1 to 5, where 1 indicates no neurological sequelae, 2 equals mild sequelae, 3 equals moderate sequelae, 4 equals severe sequelae and 5 is brain death. The CPC score was assessed at discharge.

### Statistical analysis

Descriptive data were reported as numbers (percentages) for categorical variables and means (standard deviations) or medians (interquartile ranges) for continuous variables. Group differences were studied by Chi-square for categorical variables and by ANOVA for continuous variables, and standardized mean difference (SMD) was reported. Survival was studied using the Kaplan-Meier estimator. The association between patient group and 30-day survival was studied using logistic regression, with sequentially adjusted models. Model 1 was adjusted for age and sex; model 2 was additionally adjusted for location of cardiac arrest and time to CPR; model 3 was additionally adjusted for initial rhythm. Time to CPR was defined as time to either bystander CPR or EMS CPR. A machine learning algorithm (random forest) was used for computation of relative variable importance using conditional importance. We calculated the conditional variable importance for 20 clinical variables for each of the studied groups.^19^, ^20^ The outcome was 30-day survival. The advantage of conditional variable importance is its ability to account for correlation among variables. We also studied the trends of survival for each group during the 10-year period. Statistical significance was defined as a *p*-value < 0.05. All analyses were performed in RStudio (version 4.2.0).

The study was approved by the Swedish Ethical Review Authority (Dnr. 2019–01094).

## Results

### Baseline characteristics

A total of 55,483 cases of OHCA, aged 18 years or older, were reported to the SRCR between January 1st, 2010, and December 31st, 2020; of these, 12,700 (22.9%) had obesity, diabetes, or both. Females represented 47.5% of cases in the Ob group, compared with between 31% and 37% in the remaining groups. Compared to the other groups, Ob patients were significantly younger, with a mean age of 62 (±16.6) years (*p* ≤ 0.001). The oldest patients were those with T2D, with a mean age of 75 (±10.7) years. A significantly higher burden of psychiatric disease and alcohol dependency was found in Ob patients, compared to the other groups (Table 2).

Among patients with T2D, 72.7% had heart disease as the underlying cause of cardiac arrest. The corresponding percentages in Ob patients and in the OTH group were 56.8% and 60.4%, respectively (Table 1).

**Table 1:** Baseline characteristics of the study population in relation to diabetes and obesity status.

GROUP	Other	Obesity only	Diabetes type 1	Diabetes type 2	Obesity and any diabetes	p	SMD
number	43 467	1 516	432	9 026	1 762		
PATIENT CHARACTERISTICS							
Women	14 703 (33.9)	720 (47.5)	156 (36.1)	2 807 (31.1)	642 (36.5)	<0.001	0.147
Age	68.1 (18.6)	62.0 (16.6)	64.7 (17.5)	75.1 (10.7)	68.7 (10.7)	<0.001	0.422
SOCIOECONOMIC STATUS							
Born abroad	5 995 (13.9)	230 (15.3)	42 (9.7)	1 549 (17.3)	332 (19.0)	<0.001	0.186
Country of birth or region						<0.001	0.176
Africa	286 (0.7)	11 (0.7)	2 (0.5)	79 (0.9)	5 (0.3)		
Asia	1 039 (2.4)	34 (2.3)	5 (1.2)	287 (3.2)	53 (3.0)		
EU	1 273 (3.0)	35 (2.3)	12 (2.8)	321 (3.6)	56 (3.2)		
Europe not EU	829 (1.9)	36 (2.4)	6 (1.4)	266 (3.0)	62 (3.6)		
North America	107 (0.2)	3 (0.2)	0 (0.0)	19 (0.2)	3 (0.2)		
Denmark Finland Norway Iceland	2 267 (5.3)	108 (7.2)	15 (3.5)	537 (6.0)	139 (8.0)		
Other	59 (0.1)	0 (0.0)	0 (0.0)	12 (0.1)	5 (0.3)		
Sweden	37 051 (86.1)	1 274 (84.7)	389 (90.3)	7 387 (82.7)	1 413 (81.0)		
South America	130 (0.3)	3 (0.2)	2 (0.5)	28 (0.3)	9 (0.5)		
WORK OR PROFESSION							0.275
Senior officials and senior positions	1 053 (2.7)	28 (2.0)	10 (2.6)	85 (1.0)	22 (1.3)		
Qualified officials	1 595 (4.1)	56 (3.9)	25 (6.6)	147 (1.8)	48 (2.9)		
Other officials	861 (2.2)	37 (2.6)	12 (3.1)	78 (0.9)	28 (1.7)		
Small business	1 441 (3.7)	59 (4.2)	13 (3.4)	260 (3.1)	78 (4.6)		
Supervisors and technicians	33 (0.1)	0 (0.0)	0 (0.0)	9 (0.1)	2 (0.1)		
Vocationally trained in trade service/care	1 358 (3.5)	100 (7.0)	24 (6.3)	115 (1.4)	61 (3.6)		
Vocationally trained workers	1 204 (3.1)	33 (2.3)	13 (3.4)	102 (1.2)	34 (2.0)		
Other workers	2 102 (5.4)	87 (6.1)	22 (5.8)	247 (3.0)	102 (6.1)		
Not employed	29 073 (75.1)	1 021 (71.9)	262 (68.8)	7 305 (87.5)	1 307 (77.7)		
EDUCATIONAL LEVEL						<0.001	0.233
Post gymnasium, 3 years or longer	3 584 (9.1)	110 (7.6)	28 (7.3)	533 (6.4)	94 (5.6)		
MARITAL STATUS						<0.001	0.254
Not married	9 619 (23.9)	406 (27.9)	116 (29.9)	1 279 (15.2)	351 (20.8)		
Married	18 165 (45.1)	591 (40.6)	154 (39.7)	4 115 (48.8)	803 (47.5)		
Divorced	6 513 (16.2)	335 (23.0)	71 (18.3)	1 469 (17.4)	353 (20.9)		
Widow or widower	5 939 (14.8)	121 (8.3)	47 (12.1)	1 568 (18.6)	183 (10.8)		
CAUSE OF CARDIAC ARREST						<0.001	0.369
Heart disease	23 281 (60.4)	743 (56.8)	218 (56.9)	5 824 (72.7)	1 157 (74.8)		
Overdose or intoxication	1 320 (3.4)	96 (7.3)	6 (1.6)	48 (0.6)	11 (0.7)		
Trauma or accident	1 006 (2.6)	35 (2.7)	12 (3.1)	75 (0.9)	14 (0.9)		
Pulmonary disease	2 125 (5.5)	99 (7.6)	14 (3.7)	462 (5.8)	100 (6.5)		
Suffocation	1 029 (2.7)	42 (3.2)	10 (2.6)	184 (2.3)	28 (1.8)		
Suicide	1 040 (2.7)	32 (2.4)	5 (1.3)	46 (0.6)	7 (0.5)		
Drowning	432 (1.1)	5 (0.4)	0 (0.0)	31 (0.4)	1 (0.1)		
Other	8 299 (21.5)	255 (19.5)	118 (30.8)	1 342 (16.7)	229 (14.8)		
GROUP	Other	Obesity only	Diabetes type 1	Diabetes type 2	Obesity and any diabetes	p	SMD
COEXISTING AND PREVIOUS COMORBIDITY							
Hypertension	15 587 (35.9)	881 (58.1)	256 (59.3)	6 893 (76.4)	1 568 (89.0)	<0.001	0.575
Heart failure	7 858 (18.1)	475 (31.3)	95 (22.0)	3 571 (39.6)	942 (53.5)	<0.001	0.389
Chronic ischemic heart disease	7 076 (16.3)	329 (21.7)	96 (22.2)	3 539 (39.2)	747 (42.4)	<0.001	0.315
Atrial fibrillation	7 620 (17.5)	396 (26.1)	71 (16.4)	2 830 (31.4)	648 (36.8)	<0.001	0.254
Dyslipidemia	4 530 (10.4)	295 (19.5)	115 (26.6)	3 036 (33.6)	864 (49.0)	<0.001	0.429
Angina including unstable angina	5 153 (11.9)	230 (15.2)	74 (17.1)	2 620 (29.0)	586 (33.3)	<0.001	0.279
Alcohol dependency	6 233 (14.3)	419 (27.6)	69 (16.0)	1 076 (11.9)	320 (18.2)	<0.001	0.182
Acute myocardial infarction	4 730 (10.9)	189 (12.5)	60 (13.9)	2 301 (25.5)	474 (26.9)	<0.001	0.235
Pneumonia, any	4 999 (11.5)	269 (17.7)	67 (15.5)	1 763 (19.5)	402 (22.8)	<0.001	0.143
Chronic obstructive pulmonary disease	4 562 (10.5)	302 (19.9)	35 (8.1)	1 311 (14.5)	436 (24.7)	<0.001	0.238
Phobic disorders	4 768 (11.0)	377 (24.9)	57 (13.2)	732 (8.1)	261 (14.8)	<0.001	0.209
Affective disorders	4 389 (10.1)	359 (23.7)	60 (13.9)	856 (9.5)	272 (15.4)	<0.001	0.188
Renal failure	2 829 (6.5)	201 (13.3)	65 (15.0)	1 980 (21.9)	573 (32.5)	<0.001	0.324
Cerebral infarction	3 100 (7.1)	111 (7.3)	30 (6.9)	1 423 (15.8)	247 (14.0)	<0.001	0.158
PRESCRIBED MEDICATIONS							
Anticoagulant or antiplatelet agents	13 335 (30.7)	589 (38.9)	154 (35.6)	5 235 (58.0)	1052 (59.7)	<0.001	0.333
Beta-blockers	12 485 (28.7)	589 (38.9)	154 (35.6)	4 668 (51.7)	980 (55.6)	<0.001	0.290
ACE-inhibitors or ARBs	11 962 (27.5)	549 (36.2)	194 (44.9)	4 743 (52.5)	971 (55.1)	<0.001	0.297
Diuretics	9 585 (22.1)	554 (36.5)	133 (30.8)	3 942 (43.7)	1011 (57.4)	<0.001	0.357
Lipid lowering drugs	7 893 (18.2)	336 (22.2)	153 (35.4)	4 113 (45.6)	917 (52.0)	<0.001	0.401
Calcium channel blockers	5 825 (13.4)	228 (15.0)	104 (24.1)	2 289 (25.4)	467 (26.5)	<0.001	0.185
Antidiabetic drugs	1 042 (2.4)	56 (3.7)	225 (52.1)	5 821 (64.5)	1240 (70.4)	<0.001	1.079
Antihypertensive drugs	303 (0.7)	23 (1.5)	11 (2.5)	236 (2.6)	68 (3.9)	<0.001	0.103
SPATIOTEMPORAL INFORMATION AND PRE-HOSPITAL INTERVENTIONS							
Location of cardiac arrest						<0.001	0.090
Home	30 450 (70.3)	1 105 (73.2)	313 (72.8)	6 774 (75.4)	1 353 (77.2)		
Public place	7 545 (17.4)	214 (14.2)	66 (15.3)	1 111 (12.4)	204 (11.6)		
Other places	5 292 (12.2)	190 (12.6)	51 (11.9)	1 100 (12.2)	195 (11.1)		
Witnessed cardiac arrest	27 183 (64.5)	838 (57.0)	250 (59.2)	5 991 (68.1)	1 145 (66.9)	<0.001	0.124
Witnessed by ambulance	2 856 (20.4)	124 (22.6)	27 (25.2)	768 (23.4)	185 (26.1)	<0.001	0.066
Telephone CPR	7 473 (62.7)	298 (63.9)	69 (67.6)	1 479 (61.7)	334 (64.9)	0.482	0.059
Bystander CPR	23 388 (55.9)	803 (55.1)	225 (54.1)	4 500 (51.7)	875 (51.4)	<0.001	0.049
AED connected by bystander	1 469 (6.8)	66 (7.2)	10 (5.3)	293 (6.3)	58 (5.6)	0.376	0.042
AED used by bystander	531 (37.3)	15 (23.1)	5 (50.0)	92 (32.5)	23 (40.4)	0.073	0.263
Intubation performed	12 173 (28.5)	394 (26.5)	129 (30.3)	2 433 (27.4)	434 (25.1)	0.002	0.056
Laryngeal mask placed	15 010 (59.8)	705 (66.2)	149 (65.9)	3 509 (64.3)	800 (66.7)	<0.001	0.065
Defibrillated, any	14 134 (33.8)	418 (28.7)	132 (31.2)	2 904 (33.4)	521 (30.5)	<0.001	0.056
Number of defibrillations	3.46 (3.16)	3.65 (3.23)	3.41 (3.57)	3.56 (3.14)	3.38 (3.15)	0.420	0.043
Adrenaline administered	33 530 (78.1)	1 199 (79.8)	359 (83.9)	7 181 (80.4)	1 399 (80.4)	<0.001	0.062
Amiodarone administered	5 010 (11.9)	171 (11.6)	40 (9.4)	1 062 (12.1)	185 (10.8)	0.344	0.041
CRITICAL TIME INTERVALS (min) median [IQR]							
Time from arrest to EMS dispatch	2.0 [1.0, 5.0]	2.0 [1.0, 5.0]	2.0 [1.0, 5.0]	2.0 [1.0, 6.0]	2.0 [1.0, 5.0]	0.273	0.059
Time from arrest to CPR start	3.0 [0.0, 10.0]	3.0 [0.0, 10.0]	2.0 [0.0, 10.0]	3.0 [0.00, 10.0]	3.0 [ 0.0, 10.0]	0.481	0.042
Time from arrest to defibrillation	14.0 [8.0, 23.0]	15.0 [9.0, 24.0]	16.0 [11.0, 27.3]	16.0 [10.0, 25.0]	16.0 [9.0, 27.0]	<0.001	0.085
Time from arrest to EMS arrival	13.0 [8.0, 20.0]	13.0 [8.0, 20.0]	13.0 [9.0, 19.0]	13.0 [8.0, 20.0]	14.0 [8.0, 21.0]	0.383	0.057
Time from EMS dispatch to arrival	10.0 [7.0, 16.0]	10.0 [7.0, 17.0]	10.0 [7.00, 16.0]	10.0 [7.0, 16.0]	11.0 [7.0, 17.0]	0.008	0.066
Time from arrest to ROSC	15.0 [9.0, 23.0]	15.0 [10.0, 24.0]	15.5 [10.0, 23.3]	15.0 [9.0, 23.0]	15.0 [9.0, 24.0]	0.853	0.027
INITIAL PRESENTATION							
Initial rhythm						<0.001	0.094
VF, pVT	9 277 (24.3)	251 (18.6)	80 (20.7)	1 705 (21.3)	286 (18.4)		
PEA	6 512 (17.0)	202 (15.0)	66 (17.1)	1 467 (18.3)	254 (16.3)		
Asystole	22 408 (58.7)	895 (66.4)	240 (62.2)	4 833 (60.4)	1 016 (65.3)		
Consciousness on EMS arrival at scene	4 385 (10.4)	154 (10.5)	48 (11.3)	1 002 (11.4)	235 (13.8)	<0.001	0.048
Normal breathing on EMS arrival at scene	4 807 (11.4)	168 (11.5)	58 (13.7)	1 057 (12.1)	230 (13.5)	0.003	0.071
Pulse on EMS arrival at scene	5 767 (14.0)	190 (13.3)	64 (15.6)	1 243 (14.6)	261 (15.7)	0.132	0.036
Spontaneous circulation on hospital arrival	11 668 (46.0)	340 (41.1)	112 (47.1)	2 092 (43.3)	332 (38.1)	<0.001	0.092
Consciousness on hospital arrival	2 968 (11.9)	74 (9.1)	24 (10.2)	424 (9.0)	69 (8.1)	<0.001	0.060

Data are shown as numbers (%) or mean (SD) if not otherwise specified.

CPR = cardio-pulmonary resuscitation; EMS = emergency medical service; VF = ventricular fibrillation; pVT = paroxysmal ventricular tachycardia; PEA = pulseless electrical activity.

**Table 2:** Outcomes of the study population in relation to diabetes and obesity status.

	Other	Obesity only	Diabetes type 1	Diabetes type 2	Obesity and any diabetes	p		SMD
ROSC any	14 696 (35.5)	429 (30.0)	151 (36.1)	2 852 (33.2)	505 (30.2)	<0.001		0.075
Hospitalized	9 812 (43.5)	293 (41.1)	101 (47.0)	1 593 (37.9)	278 (36.4)	<0.001		0.109
Discharged alive	4 795 (49.5)	133 (46.0)	39 (38.6)	550 (35.0)	93 (33.8)	<0.001		0.173
CPC score at discharge						0.520		0.241
CPC 1 (no sequelae)	3 076 (75.7)	83 (76.1)	29 (82.9)	342 (73.7)	62 (79.5)			
CPC 2 (mild sequelae)	603 (14.8)	19 (17.4)	6 (17.1)	87 (18.8)	13 (16.7)			
CPC 3 (moderate sequelae)	275 (6.8)	5 (4.6)	0 (0.0)	26 (5.6)	2 (2.6)			
CPC 4 (severe sequelae)	99 (2.4)	2 (1.8)	0 (0.0)	9 (1.9)	1 (1.3)			
Survival at 30 days	5 509 (12.7)	145 (9.6)	46 (10.6)	657 (7.3)	122 (6.9)	<0.001		0.102

Data are shown as numbers (%) or mean (SD) if not otherwise specified.

ROSC = return of spontaneous circulation; CPC = cerebral performance category.

Regarding coexisting conditions prior to OHCA, individuals with obesity, diabetes, or both, had a substantially greater burden of cardiovascular comorbidities. Hypertension was prevalent in 58.1% of Ob cases, 59.3% of T1D cases, 76.4% of T2D cases, and 89.0% of cases with ObD, compared with 35.9% of OTH cases. Heart failure was prevalent in 53.5% of cases with ObD, compared with 18.1% of OTH cases. A similar pattern was noted for ischemic heart disease, myocardial infarction, atrial fibrillation, renal failure, and stroke (Table 1).

### Unadjusted analyses

Cardiac arrest occurring at home was somewhat more common in patients with diabetes and/or obesity; 75.4% of the cardiac arrests in T2D patients occurred at home and 77.2% in cases with ObD, compared to the OTH group where only 70.3% occurred at home (*p* ≤ 0.001). Bystander CPR was least common in patients with ObD, 51.4%, followed by those with T2D, 51.7%, while 55.9% in the OTH group received bystander CPR. Overall, roughly one in two patients received bystander CPR. There were no significant differences in time to start of CPR between groups (Table 1).

Cases with ObD, Ob, T1D, and T2D presented less often with a shockable initial rhythm, ventricular fibrillation, or pulseless ventricular tachycardia (VF/pVT). Among ObD patients, 18.4% had VF/pVT, compared to 18.6%, 20.7%, 21.3%, and 24.3% in Ob, T1D, T2D, and OTH, respectively. Similarly, defibrillations were most common in the OTH group, in which 33.8% were defibrillated at any point in time (Table 1).

ROSC (return of spontaneous circulation) was least common in patients with obesity, with or without diabetes. Roughly 30% of these patients had ROSC during the course of resuscitation, compared with 33–36% in the remaining groups. Survival at 30 days was 12.7% in the OTH group, 6.9% in ObD, 7.3% in T2D, 9.6% in Ob, 10.6% in T1D (Table 2).

### Adjusted analyses

**Fig. 1:**
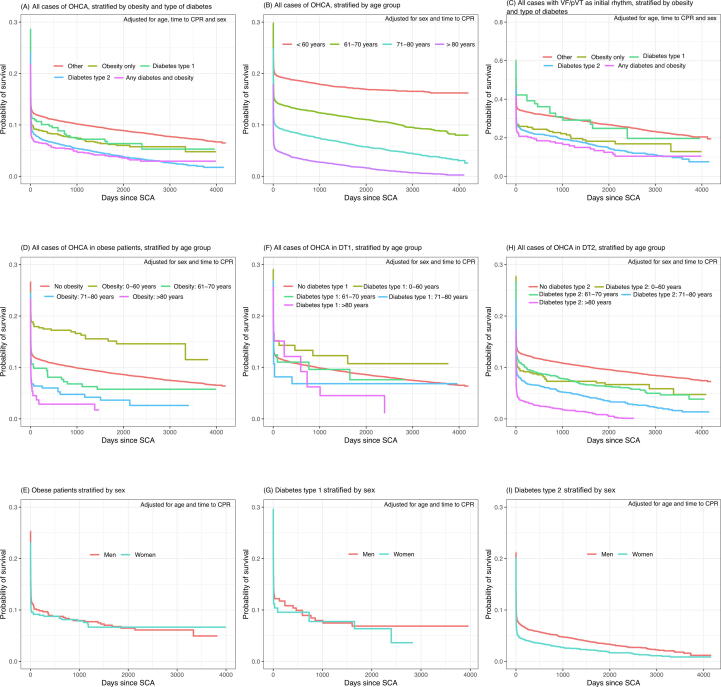
Adjusted long-term survival curves.

Fig. 1 shows survival curves adjusted for age, sex, and time to CPR for each group. Overall, the vast majority of deaths occurred within a few days of the OHCA, with clear differences in survival between groups, after which the odds ratios remained similar. The highest overall survival was noted for cases without diabetes and obesity (i.e., those defined as OTH). The poorest survival was noted for patients suffering from a combination of any diabetes and obesity (ObD) and patients with T2D. Ob patients showed survival similar to cases with T1D (Fig. 1, panel A), and this same pattern was also noted in the subgroup with shockable initial rhythm (Fig. 1, panel B). In patients belonging to groups Ob, T1D, and T2D, we noted a graded inverse association between age and survival (Fig. 1, panels C, D and E). No sex-related differences in survival were found in Ob or T1D patients (Fig. 1, panels F and G), while T2D patients showed better survival in men than women (Fig. 1, panel H).

**Fig. 2:**
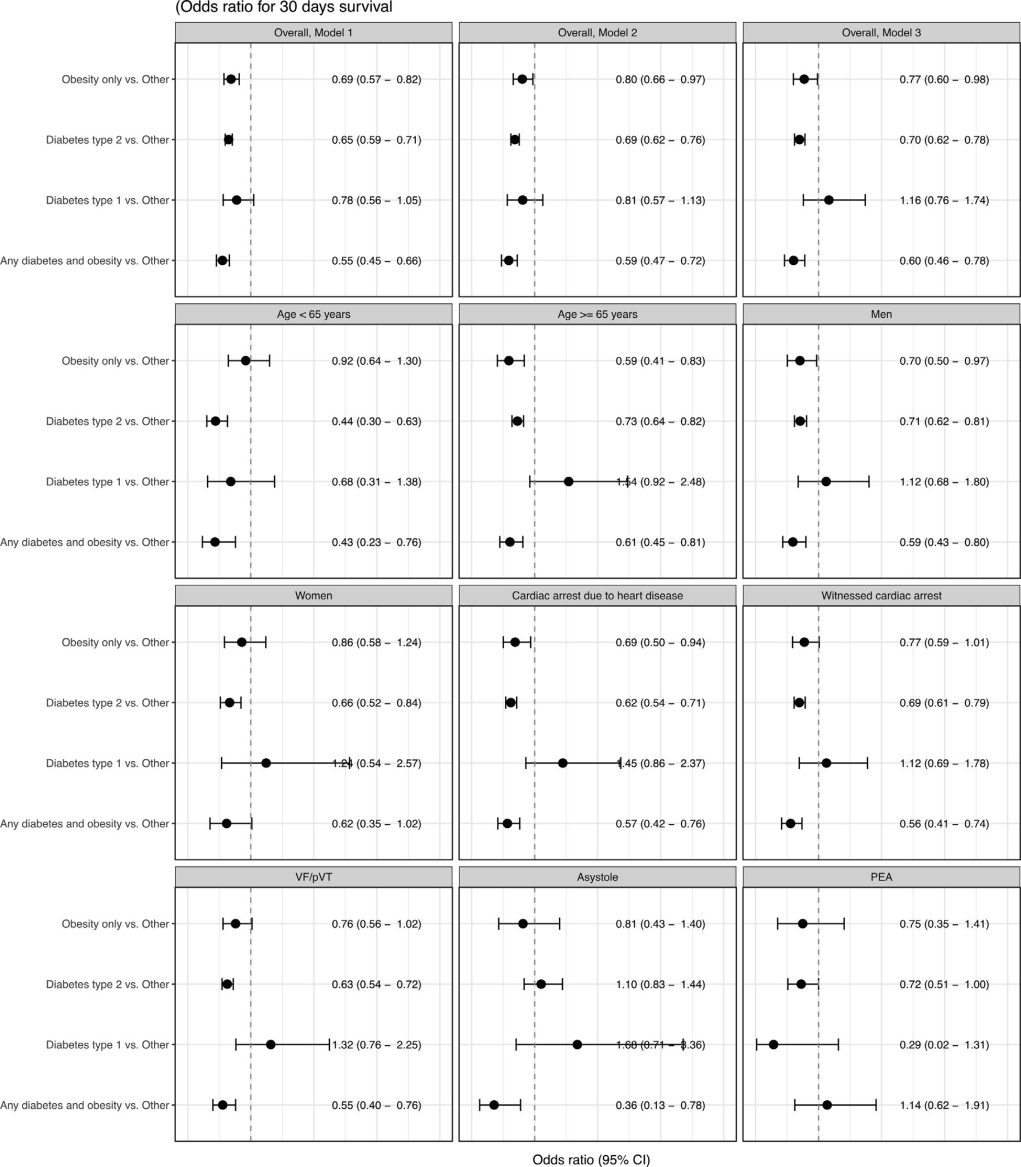
Model 1 is adjusted for age and sex. Model 2 is additionally adjusted for location of cardiac arrest and time to CPR. Model 3 is additionally adjusted for initial rhythm. VF/pVT, asystole and PEA are adjusted using model 2. Age-related OR are adjusted according to model 3, barring age. Sex-related OR are adjusted according to model 3, barring sex.

Fig. 2 shows results from the logistic regression model, in which the group defined as other (OTH) served as the reference group. Adjusting for age and sex, only cases with T1D showed survival similar to the OTH group; Odds ratios for 30-days survival were 0.69 (95% CI 0.57–0.82) for Ob cases, 0.65 (95% CI 0.59–0.71) for T2D cases, and 0.55 (95% CI 0.45–0.66) for ObD cases (Fig. 2). Additional adjustments in models 2 and 3 did not remove the statistical significance observed in model 1. The poorest survival per model 3 was noted for cases with ObD, who showed a odds ratio of 0.60 (95% CI 0.46–0.78). Similar associations were noted in the subgroups presented in Fig. 1.

### Neurological outcome

Overall, approximately 75% of the survivors achieved a CPC score of 1 at discharge. There was no difference in the probability of exhibiting CPC 1 at discharge between any of the groups (Table 2).

### Variable importance

**Fig. 3:**
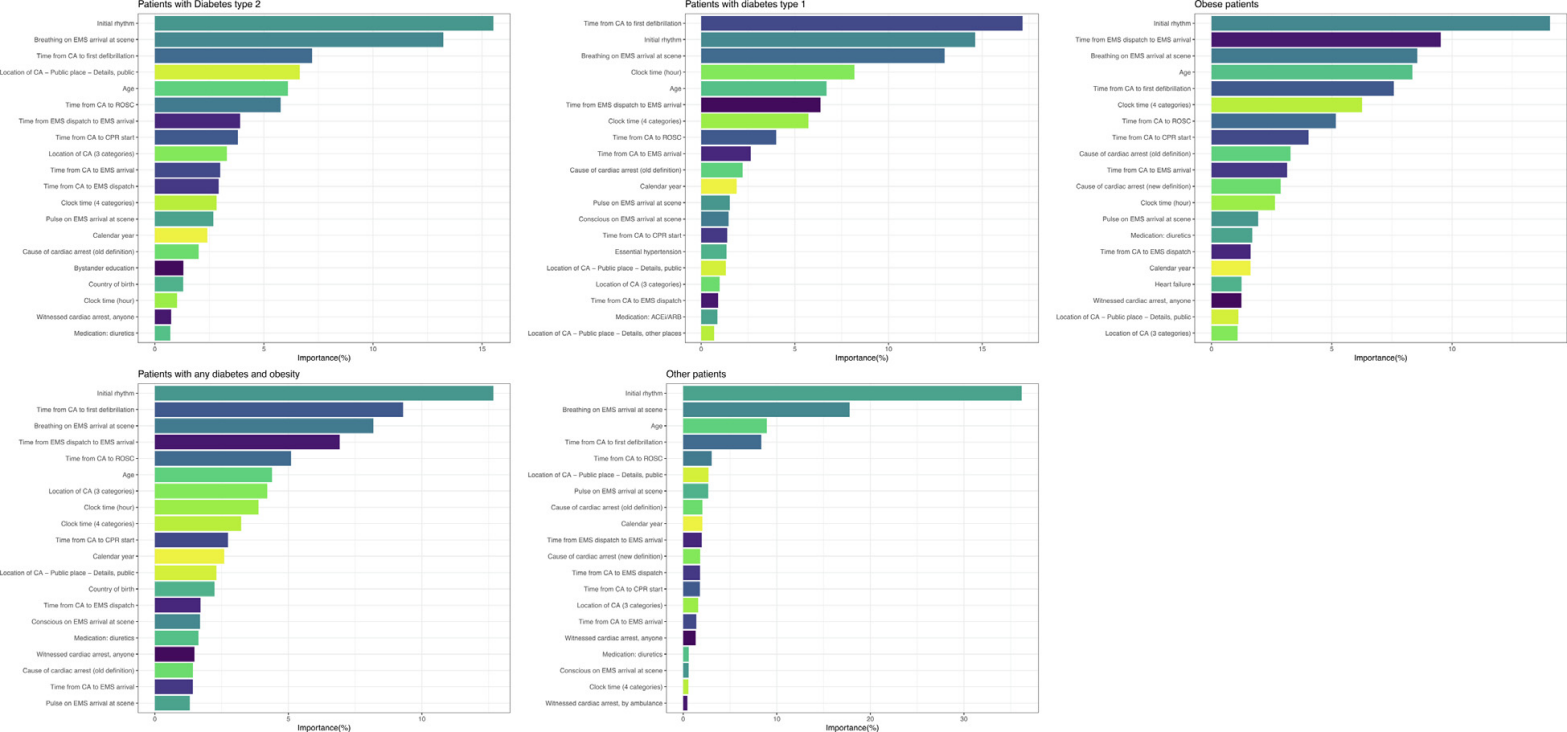
Relative variable importance for 30 day-survival in each group.

Across all groups, the strongest predictors of survival were initial rhythm, time to CPR and EMS arrival, as well as age (Fig. 3).

### Trends in survival

**Fig. 4:**
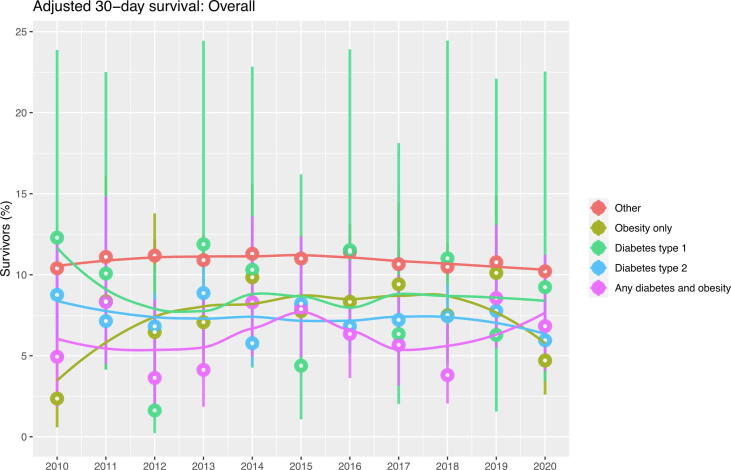
Trends in 30-day survival for each group.

There were no significant time-related changes in 30-day survival during the period 2010–2020 (Fig. 4).

## Discussion

This nationwide study focuses on individuals with obesity and/or diabetes, which is an important patient category in every aspect of cardiovascular medicine. As previously stated, an “obesity paradox” has been proposed which suggests that patients suffering from obesity have a higher chance of survival following an out-of-hospital cardiac arrest.^9^ We decided to account for diabetes diagnosis among patients as it is heavily associated with obesity.^21^ They are connected to such a high degree that terms as “diabesity” have been introduced. Evaluating the impact of the combination of diabetes and obesity is therefore highly relevant.

We show that almost one in four patients with OHCA had a history of obesity and/or diabetes and that these patients differed markedly from the other groups of patients suffering OHCA. Individuals with obesity were more frequently female, significantly younger, had less often a shockable rhythm and, in particular, concomitant diabetes and obesity resulted in halved survival compared with the general population experiencing an OHCA. Importantly, the difference in survival was not explained by the key determinants of survival in OHCA, as evident from the fully adjusted model. Regrettably, we also note that survival has not improved in any of these groups during the last decade.

Hence, obese patients – with or without diabetes – did not exhibit a favourable short- or long-term outcome after an OHCA. On the contrary, these patients were younger at the time of OHCA and exhibited a greater burden of cardiovascular comorbidities. The fact that adjusting for age, sex, initial rhythm, and time to CPR did not improve the outcome suggests that survival in these patient groups may not be improved by simply targeting the conventional resuscitation key parameters (time to EMS arrival, CPR, and defibrillation). The explanation for this remains elusive. It is not unlikely that the anatomic impact of obesity may hamper the efficiency of chest compressions as well as ability to rapidly establish an efficient airway. Current resuscitation guidelines do not mention any specific considerations (e.g., compression depth) for patients with obesity.

Even if survival was strongly affected by the presence of obesity and/or diabetes, we did not observe any difference in neurological outcome in relation to the studied groups. However, this analysis only included patients who were discharged alive; it is possible that subsequent early mortality (during the time of hospitalization) was high among those who initially survived with CPC score 2 or 3 in cases with obesity and/or diabetes, making death a competing risk in this scenario.

We noted some differences in the underlying causes of OHCA in these groups. Cardiovascular conditions were the most prevalent causes in all groups but were much less common in the group with obesity. On the other hand, obese patients showed the highest rate of pulmonary causes. The higher prevalence of chronic pulmonary disease makes this group of patients more susceptible to pneumonia and other acute lung conditions that may increase the risk for CA. This association has previously been demonstrated.^22^ Pulmonary embolism is another known cause of CA that affects obese people more frequently than non-obese people.^23^ Our data also show a higher rate of OHCA due to overdose or intoxication in the group with obesity. In addition, higher prevalence of mental illness among those with obesity suggests that obese people lead an unhealthier lifestyle overall, perhaps consuming more alcohol and other addictive substances, which is backed by the higher prevalence of intoxication as cause of the cardiac arrest in our findings.

The group with T2D was the oldest by a large margin, with a mean age of 76 years. It is therefore expected that they should have worse survival, not accounting for age. However, even when age, sex, location, initial rhythm, and time to CPR was adjusted for, they still had very poor 30-day survival. Survival in this patient category is, in fact, currently (year 2020) comparable with general survival after OHCA in the 1990s.

In analogy with the others, obese patients also presented with different initial rhythms but had the highest rate of asystole (71% vs 67% on average). This is not explained by the time to CPR, which did not differ significantly. It may, however, be explained by the differences in the cause of cardiac arrest and the different amount of witnessed cardiac arrest between the groups, as an initial rhythm of VF/pVT might deteriorate to asystole over time.^24^ It is also an indication that obesity should not be equated to coronary artery disease and myocardial infarction (which is the primary cause of shockable rhythms in OHCA).

The initial rhythm was one of the most important variables when predicting survival, together with breathing at EMS arrival and the time from the cardiac arrest to the first defibrillation. This underlines the importance of further expanding the implementation of AED which allows bystanders to give adequate care more quickly. Even though OHCA patients only present with a shockable rhythm in approximately 18–25% of cases (depending on patient group), in these occasions, a short time to the first defibrillation can make the difference between life and death.

Even though several studies have been conducted on the topic of obesity and sudden cardiac arrest, there is yet no clear evidence and consensus whether obesity improves^7^, ^8^, ^9^ or decreases^13^, ^11^, ^12^ chances of survival after out-of-hospital cardiac arrests. We believe that the greatest strength of this study, compared to previous, is our large study population and that we accounted for the presence of diabetes, both type 1 and 2, among the cases included. This gave us a broader perspective of the population and how these conditions act together and impact outcome in these patient groups.

A limitation of the study was not having data on BMI. This would have allowed us to further study the impact of not only obesity, but also overweight, and how BMI could correlate with survival in case of an OHCA. This also applies for underweight patients which might be present in the other patient groups. It follows that there will be cases with obesity and overweight in the group currently defined as *other*.

To conclude, we found evidence which was contrary to an obesity paradox. Obesity and diabetes were associated with halved survival and every effort should be directed at improving survival in this relatively young patient group.

## Credit author statement

A.H and P.P conceived the study. A.H and P.P drafted the first manuscript and performed all statistical calculations. All authors reviewed, commented, and revised all versions of the manuscript.

## Declaration of Competing Interest

The authors declare that they have no known competing financial interests or personal relationships that could have appeared to influence the work reported in this paper.
